# Missing the Wood for the Wrong Trees: On the Difficulty of Defining the Complexity of Complex Problem Solving Scenarios

**DOI:** 10.3390/jintelligence5020015

**Published:** 2017-04-13

**Authors:** Jens F. Beckmann, Natassia Goode

**Affiliations:** 1 School of Education, Durham University, Leazes Rd, Durham DH1 1TA, UK; 2 Faculty of Arts, Business and Law, Centre for Human Factors and Sociotechnical Systems, University of the Sunshine Coast, 90 Sippy Downs Drive, Sippy Downs, QLD 4556, Australia; ngoode@usc.edu.au

**Keywords:** complexity, difficulty, Person, Task and Situation, system characteristics, DYNAMIS, complex problem solving

## Abstract

In this paper we discuss how the lack of a common framework in Complex Problem Solving (CPS) creates a major hindrance to a productive integration of findings and insights gained in its 40+-year history of research. We propose a framework that anchors complexity within the tri-dimensional variable space of Person, Task and Situation. Complexity is determined by the number of information cues that need to be processed in parallel. What constitutes an information cue is dependent on the kind of task, the system or CPS scenario used and the task environment (i.e., situation) in which the task is performed. Difficulty is conceptualised as a person’s subjective reflection of complexity. Using an existing data set of *N* = 294 university students’ problem solving performances, we test the assumption derived from this framework that particular system features such as numbers of variables (NoV) or numbers of relationships (NoR) are inappropriate indicators of complexity. We do so by contrasting control performance across four systems that differ in these attributes. Results suggest that for controlling systems (task) with semantically neutral embedment (situation), the maximum number of dependencies any of the output variables has is a promising indicator of this task’s complexity.

## 1. Introduction

Over the past four decades, research conducted under the umbrella of complex problem solving (CPS) has produced a substantial body of studies that have addressed a wide range of research questions. These include, but are not limited to, validity-related questions focussing on CPS’ relationship to other constructs, with reasoning ability being a particularly prominent candidate [1,2]. Another research focus has been the study of cognition, including decision-making, knowledge acquisition, or learning [3]. Again, other researchers have used CPS scenarios as assessment tools to predict occupational success [4,5] or academic attainment [6]. CPS scenarios have also been used as training or educational tools in a wide range of domains [7,8].

It could be argued that despite, or maybe because of, its prolificacy, CPS research is still lacking a coherent framework that would allow the integration or synthesis of the multitude of findings. The research field (and CPS scenarios in particular) is much more diverse than the commonly used label CPS seems to suggest. In other words, whilst CPS research keeps producing many jigsaw pieces, we are occasionally left wondering whether some of these pieces even belong to the same puzzle. This perceived lack of coherence presents a major challenge to the positioning of CPS within the realm of the human intellect (or even beyond, when it comes to non-cognitive dimensions). The answer to the question of where and how to position CPS within a nomological network that is embedded within cognitive sciences—be it conceptually, methodologically or psychometrically—is fundamental to its usefulness as a tool for research, learning or assessment. 

With this paper we address barriers to the productive integration and synthesis of research findings in the field of CPS by first briefly reflecting on some factors that potentially contribute to the lack of coherence within the field. We then introduce a framework for comparing CPS scenarios to one another with regard to their complexity. In the second part of this paper we test some of the predictions derived from this framework empirically. 

### 1.1. A (Complex) Problem

One potential hindrance to coherence within the field is that the term CPS is used with at least four different meanings. CPS is used: (1) as a label for a research paradigm studying information processing, decision-making, causal reasoning, etc. (e.g., [3]); (2) as a label for various ability constructs, e.g., the ability to deal with uncertainty, that might or might not be related to intelligence (e.g., [2]); (3) in the context of international large scale assessment exercises, CPS is often discussed as a skill or competence (e.g., [9]), or (4) simply as a descriptor for a class of behaviour exhibited while dealing with a specific kind of challenges.

Another challenge to the integration and synthesis of research findings is related to the multitude of tools used, i.e., computerised scenarios or microworlds. In general terms, those tools can be described as dynamic systems of inputs (variables that the problem solver can change) and outputs (outcomes that are generated by the system), which are linked via causal structures of varying degrees of determinism that can be described algorithmically. The systems are considered to be “dynamic” because the values of the outputs change in response to the problem solvers’ actions, as well as independently over time [10,11]. 

CPS, when used as a descriptor for a class of behaviour (cf., the fourth meaning of CPS mentioned above) that is related to problem solving, often involves two distinct tasks. First, problem solvers must acquire knowledge about the underlying structure of the system. To that end, they are often given the opportunity to freely manipulate the values of the input variables and draw inferences about the causal structure based on the observed changes in the output variables. Second, problem solvers are subsequently asked to manipulate the values of the input variables over a certain number of trials in order to either reach or maintain given target values in the outputs (i.e., system control). 

Complex problem solving scenarios, or CPS systems, which fit this rather generic definition, can differ in many ways. For instance, scenarios differ in their user interfaces. Some use graphical representations of system states [12], some use numerical information [13], and others use a combination of both [14]. Despite the potential effects the format in which information is presented might have on the information processes involved in complex problem solving, it seems that decisions regarding the interface are not always given the attention they deserve. Whilst the interface designs of CPS scenarios employed in the early days were constrained by the limited graphical capabilities of computers available back then, scenarios of more modern provenance sometimes give the impression of overindulgence in the richness of graphics options modern IT technology can offer with consequences that seem not always to have been thoroughly reflected upon. This is not to say that there is a lack of effort or established knowledge regarding interface design in instructional contexts. The issue rather seems to be that the development of CPS tools used for research, learning or assessment purposes has not always benefitted from the insights gained in the fields of Cognitive Ergonomics and Instructional Design.

CPS scenarios might also differ regarding the semantic contexts in which they are embedded. The use of semantically rich cover stories and accordingly labelled variables often aims at creating the impression of “real-world” relevance to ensure problem solvers’ engagement or foster “ecological validity”. Others, however, use abstract scenarios with the intention of minimising the uncontrollable effects of prior knowledge, beliefs or suppositions [15,16]. 

Both the way in which information is presented and how the problem solver interacts with the system have an impact on (a) what kind of information is encoded by the problem solver, (b) how information is internally represented by the problem solver, (c) the kind of cognitive manipulations performed on those internal representations, and ultimately (d) the performance patterns shown when dealing with those scenarios. 

We argue that insufficient awareness of the potential effects of these and other “surface features” of systems used in CPS research contributes to an undifferentiated discussion of the term that is central to this research paradigm, namely complexity. The conceptual ambiguity regarding the term complexity has been and continues to be a major roadblock to progress in CPS research. The main objective of this paper, therefore, is to conceptually contribute to a better understanding of complexity in the context of CPS. We aim to achieve this in two steps. First, we introduce a conceptual framework with complexity at its centre. Second, we test some of the assumptions derived from this framework empirically. This, we hope, will re-ignite reflections and discussions about what we do in our CPS-related research and provide suggestions as to how to better integrate its findings.

### 1.2. The Tripartite Framework of Person, Task and Situation (PTS)

In general terms, the psychologically orientated study of learning and problem solving takes place in the context of three categories of variables: person variables, task variables and situation variables. *Person* variables comprise what can be described as genuine psychological concepts such as ability, motivation, knowledge, skill, etc. The definition of *task* is slightly less straightforward. As discussed in Wood [17] (p. 61), a *task* can be defined from four different perspectives^1^ [18,19]. (1) The focus of the “task qua task” labelled perspective is on the physical characteristics of the stimuli that need to be processed as part of performing a task. (2) A *task* can also be defined by the kind of behaviour that needs to be exhibited in order to meet specified levels of performance, forming the “task as behaviour requirement” perspective. (3) In contrast, a “task as behaviour description” perspective focuses on a post hoc description of what people “typically” do when performing a task. (4) Another perspective describes a “task as ability requirements”. Both perspectives (3) “the task as behaviour requirement” and (4) “task as ability requirement” are limited in their conceptual usefulness due to their post hoc and circular nature, which, in the context of CPS, translates into “complex problem solving is what people do when they solve complex problems”, or “complex problem solving requires the ability to solve complex problems”, respectively. We, therefore, focus in out attempt to conceptualise task related complexity on the (1) “task qua task” and the (2) “task as behaviour requirement” perspectives. 

The third category in the PTS framework is the situation. *Situation* variables represent the circumstances in which a given task has to be performed (i.e., the task environment, as discussed by Newell and Simon, 1972, p. 55, [20]). In complex problem solving this relates to aspects such as the semantic embedment of a CPS scenario, or range restrictions for input variables as part of the user interface, to name only two examples. Beckmann et al. [21] give an example where relatively minor changes in situational variables help to ensure that problem solvers execute the task of knowledge acquisition more systematically, which leads to positive effects on their control performance. 

The perspective that combines a task, in its sub-facets of “task qua task” and “task as behavioural requirement”, and a situation as a “task environment” makes complex problem solving a requirement for cognitive behaviour that is directed towards processing stimulus characteristics or elements of information that are presented in a specific form. More specifically, the task of controlling a system, for instance, translates into “causing the values of a certain number of output variables to change to the set of goal states by appropriately manipulating a certain number of input variables by utilising acquired knowledge about the system”. 

Based on this conceptualisation, we can identify two general sources of complexity in CPS: The *complexity of a task qua task*, which is determined by system characteristics and the *situation complexity*, which is determined by the circumstances in which the given task is to be performed. These sources of complexity are interdependent insofar as the specifics of the performance requirement (i.e., what the problem solver is instructed to do) determine which of the system characteristics constitute information cues that need to be processed, which subsequently informs the cognitive behaviour to be executed. At the same time, situation characteristics, since they determine whether or in what form relevant information cues are available, also contribute to the overall complexity. The interplay of these two sources of complexity is determined by the task given to the problem solver (i.e., the task as behaviour requirement) in three ways: (1) The set of cognitive acts required to execute different tasks (e.g., knowledge acquisition vs. system control) will differ, and so will their complexity, despite using the same system and presenting it in the same format. (2) The complexity of executing a task (be it to control the system, or to acquire knowledge about its structure), which requires certain cognitive acts, is also affected by the physical attributes of the system (e.g., number of variables or quality of relationships between variables). (3) Different task environments (e.g., graphical interface, or semantic embedment) are likely to trigger different cognitive behaviours, despite being confronted with the same task (e.g., to control the system) and using the same system, which subsequently will result in differences in complexity. 

Both system characteristics and situational characteristics are constituents of complexity. Whilst complexity is conceptually linked to the task (qua task or as behaviour requirement) and the situation, the concept of *difficulty* is linked to the third variable in this PTS framework, i.e., the person. Performance scores are therefore indicative of the difficulty a person experiences whilst dealing with the demands imposed by the task and situation. This makes difficulty the subjective reflection of the complexity of a given task presented under given circumstances. Difficulty and complexity cannot be used interchangeably. In fact, the necessary conceptual dissociation of complexity and difficulty enables us to identify potential problems with the design of the research tools we have used. For instance, if a task (presented in a situation) is completed with fewer difficulties than complexity would have led us to expect (i.e., better performance), this may suggest that the problem solvers were not performing the sequence of cognitive steps we anticipated. This can occur, for instance, when problem solvers are able to utilise previously acquired schemata, allowing them to chunk information cues instead of processing them separately [17]. Presenting a complex, dynamic system embedded in a semantically rich context, for instance, might trigger such a situation. If problem solvers can rely on prior knowledge, they can circumvent the demand of performing the cognitive acts related to a systematic exploration of the system’s causal structure. Such a situation would be an example of where well-intentioned implementation of situational characteristics (e.g., to make it “more real or relevant”) has unwittingly created a validity threat (see [22,23] for examples), as performance scores under these circumstances might not necessarily be indicative of the ability to acquire *new* knowledge in dynamic environments. In Messick’s terms, such situations create the validity threat of “construct-irrelevant easiness” [24]. Analogously, situational features could introduce unnecessary layers of complexity that subsequently might impede the employment and execution of the anticipated sequence of cognitive steps, jeopardising validity through “construct-irrelevant difficulty” [24]. As an example, the unavailability of a learner’s log that would have allowed the problem solver to access previous decisions and their associated consequences might prevent the integration of new insights into an existing knowledge base or schema and its subsequent modification as a result of learning using a complex, dynamic system. Unless intended, the lack of crucial information cues is likely to cause a situation where (theoretically pre-determined) complexity and (empirically observed) performance can be misaligned. 

Constellations in which control performance appears to be better than expected (based on knowledge scores), in combination with low correlations between knowledge and control performance, have occasionally encouraged post hoc speculations regarding the acquisition of implicit knowledge [25]. This, of course, can be problematic. Dissociations between knowledge and control performance can alternatively be explained by an intervention heuristic previously described as “ad hoc control” [26] (p. 195). The rationale behind this heuristic is, simply put, interventions that cause the system to deviate further from the goal states will be (over-)compensated for with the subsequent intervention and interventions that bring the system closer to the goal states and thus are likely to be repeated. Such an intervention-by-intervention optimization does not require system knowledge yet might result in acceptable levels of control performance. 

Within the three-dimensional variable space of Person, Task and Situation, we have argumentatively established that the task and the situation serve as distinct contributors to complexity and we have conceptualised difficulty as a person’s reflection of complexity. Now, since we have clarified *where* to look for sources of complexity, we turn to a discussion of *what* contributes to system complexity and situational complexity and ultimately to task complexity. Such reflections will inform our approaches to their respective operationalization.

### 1.3. Characteristics of Task and Situation and Their Contributions to Complexity 

The problem of defining complexity is not confined to research with dynamic systems, and there exists an extensive literature on how the complexity of cognitive tasks might be conceptualised and measured. One approach, known as Relational Complexity Theory (RCT), is that complexity can be conceptualised as a function of the number of relations and sources of variation that must be integrated in order to formulate a solution to a (sub-)task [27]. Decrements in performance related to complexity are explained by constraints on the capacity to process information in parallel, which has long been recognised as a critical constraint on human performance in general [28]. Findings show that increases in the number of relations that must be processed in parallel in reasoning tasks consistently lead to increases in task difficulty [27,29,30,31]. This conceptualisation of complexity is closely related to the concept of element interactivity within Cognitive Load Theory (CLT, e.g., [32,33]). Element interactivity is the extent to which information elements in learning material interact and must be processed in parallel in order to be learned. Element interactivity is seen as the main contributor to the overall cognitive load of a task [34,35]. This perspective applied to CPS would lead to the expectation that systems in CPS with a higher level of elements (i.e., variables or relationships between them) that must be processed *in parallel* are more complex. In the context of intelligence research, Stankov’s notion of complexity [36] is based on the premise that tasks with higher levels of complexity place greater demands on cognitive resources. Therefore, a monotonic increase in the correlation between task performance and cognitive ability measures should be seen as an indication of increasing levels of complexity (see also [37]). A possible adoption of this view for CPS is challenged by two issues: First, such an argument results in a psychometrically grounded operational definition of complexity, and second, correlation patterns between CPS performance scores and cognitive ability measures are in and of themselves frequently part of the validity debate. Simply put, too high a correlation might raise concerns regarding CPS’s distinctiveness from traditional approaches in terms of ability to measure cognitive abilities; too low a correlation, on the other hand, seems to indicate low levels of complexity, and, of course, one would be hard pressed to specify what constitutes too low or too high.

In the context of CPS research, a range of studies have looked at the links between more or less readily quantifiable system characteristics such as numbers of variables or numbers of relationships and performance scores. For example, the number of relationships in a system has been identified as being predictive of performance (e.g., [14,38,39]). Other research looked at various combinations of number of variables and the number and kind of relations that exist among those variables [38,40,41,42,43,44]. Other studies, however, explored links of a range of system characteristics with IRT-based estimates of item difficulties [45,46]. 

An overall consensus seems to exist in that a higher count of any of those system characteristics results in higher levels of complexity, which in turn is thought to make it more difficult to acquire structural knowledge and/or to control the outcomes of the system [14,40,41,47].

By taking performance scores as indicators of the level of difficulty problem solvers have experienced, those system features that are correlated with performance scores are declared contributors to complexity. In other words, if the decision regarding what constitutes a contributor to complexity is exclusively based on a psychometric analysis of correlations, difficulty and complexity are conceptually indistinguishable. Post hoc rationalisations of correlation patterns as to why system feature X is but system feature Y is not a complexity indicator provide little orientation. Maybe this is the main reason for the interchangeable use of complexity and difficulty in the CPS literature. 

The application of the proposed Person, Task and Situation framework to CPS scenarios (a) enables a conceptual differentiation of complexity and difficulty, (b) brings situational characteristics into the conceptual focus, and (c) conceptually accommodates the fact that different system features gain complexity relevance depending on the instructed task and the given situational characteristics. It therefore facilitates a necessary shift from a predominantly psychometric outlook on complexity towards a more conceptually underpinned perspective. 

A generalised orientation on the number of variables (NoV) or number of relationships (NoR), as an example, stands in contrast to the Person, Task and Situation framework outlined above in at least two ways. First, such a focus seems to ignore that what constitutes an information cue in one task context (such as to acquire structural knowledge about the causal structure of a system) might be complexity irrelevant in another (e.g., controlling the system), and vice versa. Second, a focus on the *total* numbers of variables or relations tends to ignore that what constitutes an information cue that contributes to complexity is something that has to be processed in parallel. Making decisions regarding inputs to control a system (i.e., control interventions) rarely requires the consideration of all relations or variables in parallel. The decision-making processes for identifying the appropriate inputs to determine the underlying structure of the system (task of acquiring knowledge), or inputs to reach and maintain a given goal state (task of controlling the system), are sequential. For instance, to reach and maintain a given goal state, problem solvers must decide how to alter each input variable in turn. Hence, we argue that an operationalisation of complexity based on the *total* number of variables or relations within a system would be misleading. 

**Figure 1:**
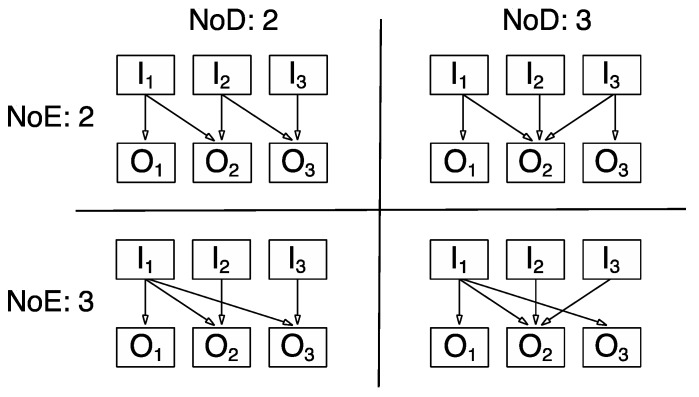
Examples of systems that differ in number of effects (NoE) and number of dependencies (NoD) whilst having the same number of variables (NoV, i.e., three inputs, I_1_, I_2_, I_3_ and three outputs, O_1_, O_2_, O_3_) and the same number of number of relationships (NoR).

With regard to connectivity, i.e., the “dependency between two or more variables” [3], (p. 73), which seems more in alignment with the above framework, we propose a modification of its original operationalization that (a) takes into account the sequential nature of decision making processes when dealing with CPS scenarios, and (b) reflects the task specificity of system features in terms of their contribution to complexity. As an example of the latter, the task (as behaviour requirement) of acquiring knowledge about the causal structure of a system is expected to result in *effect knowledge* (e.g., “What happens if I change input ‘A’”?), whilst the task of controlling a system builds on *dependency knowledge* (e.g., “Which of the inputs do I have to change to cause ‘X’ to reach its goal state?”). In the context of a knowledge acquisition task, we propose that the maximum number of effects (NoE) any of the input variables have on any of the output variables constitutes a performance-relevant system feature of complexity. In the context of the task of controlling the system, we propose that the maximum number of dependencies (NoD) any given output variable has from different input variables constitutes a performance-relevant system feature of complexity. As an illustration, Figure 1 provides an example of how NoE and NoD can vary independently across systems with the same NoV and NoR.

Studies that systematically studied the effects of any of the abovementioned system features in combination are rare (see [48] as an example). Funke [49,11] and Kluge [14] manipulated in their studies the number of relations (NoR), which also resulted in changes in the number of dependencies (NoD). Thus, the results of these studies could also be interpreted as evidence that the connectivity implied by the goal states provides a good indicator of system complexity in relation to knowledge acquisition and system control. However, obviously, it cannot be determined whether these results are attributable to the number of relations, the connectivity implied by the goal states or both. The current study will attempt to determine whether either of these system features have an independent effect on system complexity.

In sum, it seems plausible that the number of relations (NoR) and the number of dependencies (NoD) should impact upon the information processing demands of knowledge acquisition and system control, although for different reasons. Systems with larger numbers of relations, but similar levels of connectivity, require more information to be processed sequentially during knowledge acquisition and system control. Systems with higher numbers of dependencies (NoD) but the same number of relations require problem solvers to process larger amounts of information in parallel during system control, but probably less so for knowledge acquisition. For numbers of effects (NoE) we would expect the reverse. 

In the remainder of this paper we are focussing on testing some of the assumptions derived from the proposed complexity framework empirically. Our focus will be on the effects of the number of variables (NoV), the number of relations (NoR) and the number of dependencies (NoD) on control performance. To this end, we use an existing dataset collected for a study reported in [50]. This dataset provides a useful opportunity to contrast the effects of the abovementioned system features (task qua task) on control performance across four different systems whilst keeping both the task (i.e., task as behaviour requirement, in this case to control the respective system) and the situational features (i.e., the same start-goal discrepancy and the same semantically neutral embedment) constant. Another crucial feature of this design is that person variables—on average—also do not vary systematically between the experimental groups. This constellation enables us to determine the role of those selected system features (i.e., NoV, NoR, NoD) as complexity factors in CPS by analysing the link between systematic differences in these system features and performance outcomes shown. 

## 2. Materials and Methods 

*Participants*: Two hundred ninety-four students at the University of Sydney participated for course credit (*M* = 19.63 years, *SD* = 3.60, range: 17–55 years). Seventy per cent of the participants were female. Seven participants failed to complete all of the tasks, so their data were excluded from further analysis. 

**Figure 2:**
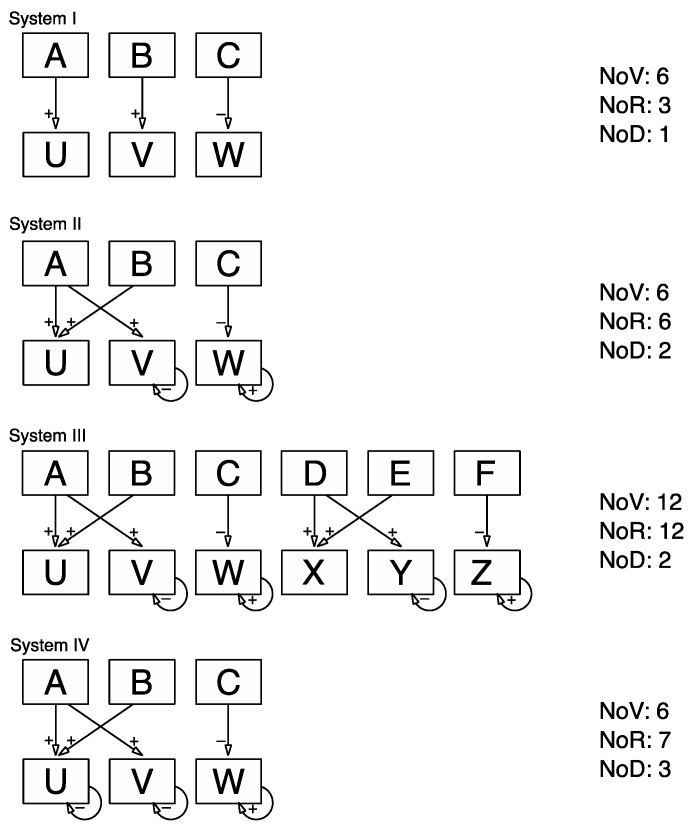
Causal structures of the four systems used in this study.

*Materials*: The analysis builds on a between-subjects design with four levels of system complexity. We contrast the effects of three different system features across four systems. To this end, we use a system comprising three input variables and three output variables that are linked by a total of six relationships (see system II in Figure 2 for its causal diagram and Appendix A for the corresponding set of structural equations) as reference point. This system, from now on referred to as system II, has been used previously in numerous studies in relation to a wide range of research questions [12,15,16,26]. In the version used here, variables are labelled “A”, “B” and “C” for the inputs and “U”, “V” and “W” for the outputs. The user interface is in a non-numerical graphical format. We manipulated the causal structure to create three additional systems. In contrast to system II, the number of relationships was reduced to three for system I, whilst the number of variables remains the same. For system III the number of variables as well as the number of relationships were doubled in relation to the reference system II, resulting in a system with six input variables and six output variables that are linked via 12 relationships. In comparison to system II, system IV had one additional relationship whilst keeping the number of variables the same (see Figure 2 for a diagrammatic representation of the four systems used in this study).

*Procedure*: Participants were randomly allocated to one of the four complexity conditions. Each group had to first acquire knowledge about the underlying causal structure of their respective complex, dynamic system and then control it by reaching and maintaining a goal state. The start and goal state were the same across the four systems. Problem solvers were not informed about the goal state prior to the actual control phase. To check whether the planned between group comparisons are not challenged by pre-existing systematic differences in the ability to deal with complexity participants had to also complete a 20-item short form of the Advanced Progressive Matrices. 

Using systems that differ with regard to features that potentially contribute to the complexity of a control task comes with a particular challenge as those features might be relevant in terms of a knowledge acquisition task but not for a control task (and vice versa). Also, the use of systems with differing levels of complexity ultimately leads to systematic differences in knowledge acquisition scores, e.g., it will be easier to acquire system knowledge in systems with low levels of complexity than it is in systems with higher levels of complexity. Consequently, superior control performance in low complex systems is not necessarily an indication of the complexity of the control task itself but could merely be a complexity effect that has been carried forward from the exploration task (i.e., knowledge acquisition). In order to deal with the conceptual challenge of disentangling knowledge acquisition from the control task and the technical challenge of potential range restrictions (i.e., ceiling effects in low complexity and floor effects in high complexity) we randomly subdivided each of the four complexity sub-samples into three groups. Instead of allowing problem solvers to freely explore the system, which would have resulted in a spectrum of individual differences in system knowledge, we exposed participants to three variants of a controlled instruction regarding system knowledge. Participants worked under three information conditions to ensure comparability across the four complexity conditions in regard to their starting point (i.e., “levelling the playing field”) whilst maintaining a maximum spread of “preparedness” for the task of controlling the system. During this instruction phase participants were shown a recording of a number of intervention trials with an accompanying narration, while a causal diagram was constructed on screen to record this information. The information remained on screen during the control cycles. In the complete information condition, a strict VONAT approach (i.e., Vary-One-or-None-At-a-Time, [16], p. 279) was realised: On the first trial the inputs were all set at zero, so that any autonomic changes in the outputs could be detected. Subsequently, Input A was increased to maximum while the other input variables were set at zero, and then on the next trial it was decreased to minimum while the other input variables were set at zero. This was repeated for each input variable, so that the effect of each input on the outputs could be clearly observed. After each trial, the narrator described how the inputs had been altered, how each of the outputs had changed, and how this reflected the underlying structure of the system. Participants in the partial information conditions received similar instructions. However, they were not shown the effect of input B on the outputs, nor did their causal diagram reflect this piece of information. Instead, on these trials all the inputs were increased to maximum, and then all the inputs were decreased to minimum. In the no information condition, multiple inputs were varied on each trial. In this condition, at the end of each trial the narrator explained how the outputs had changed, but did not make any inferences with regard to the structure of the system, and a causal diagram was not developed on screen. 

Participants then completed a test of structural knowledge. For each item of the structural knowledge test, participants were shown the input variables in a particular configuration. They then had to predict for each output variable whether it would increase, decrease or stay the same as a result of this intervention. This method of assessing structural knowledge has been tried and tested in a number of studies in the past [1,15,51,52].

**Figure 3:**
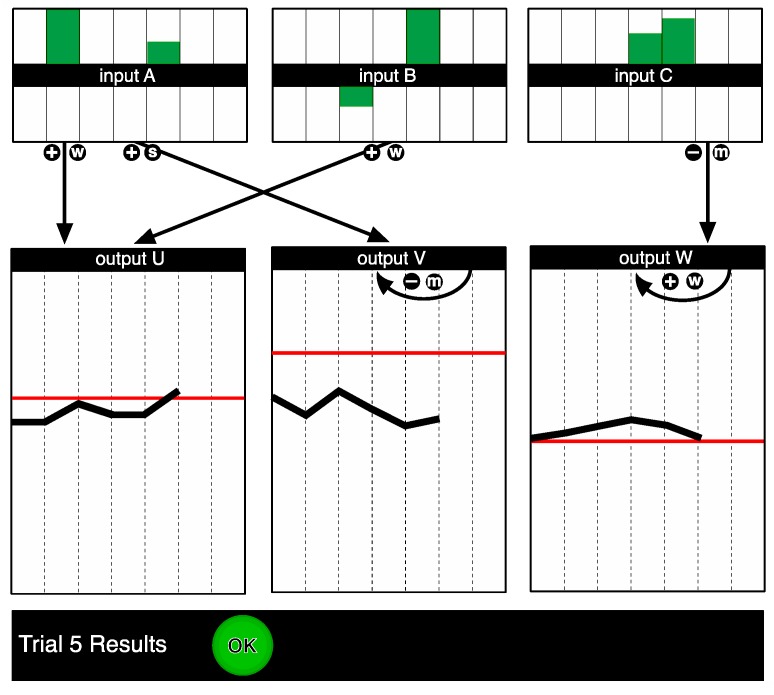
Screenshot of system II as it was presented in the complete information condition for the control phase. The goals are indicated as red lines on the graphs for the output variables. The underlying structure of the system is represented on screen as a causal diagram, where the arrows represent the relationships between the variables, while the positive and negative signs denote the direction of the effect, and the letters the relative strength. In this example, Input A, B and C were increased on Trial 5. As a result, Output U increased, Output V increased, and Output W decreased, as depicted in the output variable windows.

After completing the knowledge test participants had to control the system, i.e., they had seven trials to bring the outputs to a predefined set of values (of which they had no knowledge during the instruction phase). These goal states were indicated as lines on the output graphs. During the control cycle, the structural information appropriate to each condition was available on screen (see Figure 3).

### 2.1. Operationalisation 

*Structural knowledge.* For each item in the knowledge test, the predicted value of all output variables had to be in the correct direction in order to receive one point. Partial credit was not awarded. The scores were transformed into percentages.

*Control performance.* Control performance was calculated by determining the Euclidean distance between the intervention vector (i.e., values entered for the input variables) made by the participant and the vector of optimal intervention (i.e., inputs that would have brought the outputs at or closest to the goal states^2^) for each trial. Finding the correct control intervention can be seen as navigating a problem space. Different systems differ with regard to the size of their problem space and so do different start-goal discrepancies used for the same system. In order to allow for comparison of performance scores (i.e., the trial by trial deviation from the optimal) across different systems and/or different start-goal discrepancies scores need to be standardised against the size of the problem space of the respective system (or start-goal discrepancy). This was realised by dividing the trial specific deviation scores by the trial specific difference between the vectors of pessimal and optimal intervention inputs (see Formula (1)).

(1) $$D=\frac{1}{m}\overset{m}{\underset{t=1}{\sum }}\left\{1-\lceil \frac{\sqrt{\sum_{i=1}^{k}{\left(optimal_{ti}-actual_{ti}\right)}^{2}}}{\sqrt{\sum_{i=1}^{k}{\left(pessimal_{ti}-optimal_{ti}\right)}^{2}}}\rceil \right\}$$

*m*: number of trials in control cycle (seven for all conditions).

*k*: number of input variables (three in systems I, II, IV; six in system III).

As a result, control performance scores represent the averaged (across the seven control trials) deviation of the actual from the optimal intervention relative to maximal possible deviation for each and every trial. Their theoretical range is from 0 (worst possible, i.e., pessimal) to 100 (i.e., optimal). Such scaling enables a fair comparison of performances shown in systems of different complexity.

*Reasoning ability*: The percentage of correct responses on an abridged version of the Raven’s Advanced Progressive Matrices (APM, [53]) was used as an indicator of reasoning ability. This version of the APM included 20 items from the original 36-item test, created using the odd numbered items plus two additional even-numbered ones from the most complex items (i.e., items 34 and 36). 

### 2.2. Analysis Strategy

To test the role of selected system features as performance-relevant contributors to the complexity of controlling a complex, dynamic system, we employed a regression analytic approach with dummy coded contrasts. For these planned contrasts we use system II as the reference system, as it represents the “middle ground” with its constellation of number of variables (NoV), number of relations (NoR) and number of dependencies (NoD) in the context of the four systems used in this study. 

The pattern in which the four systems differ with regard to the three system characteristics outlined above provides an opportunity to use planned contrasts to establish whether these system features can be considered contributors to the task of controlling a system. For instance, system III does differ from system II (as the reference system) with regard to the number of variables (i.e., 12 vs. 6). If the number of variables (NoV) was a system characteristic that contributes to the complexity of controlling a system we would expect the β-weight related to the contrast between system II and III to be significantly different from zero (in fact we would expect it to be negative, in this case). 

In CPS, it is (a) available system knowledge and (b) the ability to use it that enables a problem solver to deal with the complexity of the task to control a system. The success of it is reflected in the control performance level. Control performance scores are in this regard indicators of the translation of complexity into difficulty. The “typical” CPS routine, i.e., control phase following a knowledge acquisition or exploration phase, however, imposes a challenge to the planned analyses. The challenge is that control performance in systems of different complexity is confounded by the knowledge problem solvers have acquired (i.e., it is easier to acquire knowledge in a less complex system). In an attempt to disentangle the effects of complexity differences in regard to the exploration task from those in relation to the control task, we included knowledge scores as covariates into the regression on system control performance. 

Each of the three complexity candidates (i.e., NoV, NoR, and NoD) is associated with a distinct result pattern (see Table 1). As an example, if the number of variables (NoV) were a performance-relevant contributor to the complexity of a control task we would expect contrast 1 and 2 not to be significant and contrast 2 to be significantly negative. The predicted result pattern that matches the empirically obtained one indicates the relative importance of its associated system characteristic in terms of complexity. 

## 3. Results

Table 2 provides an overview of the descriptive statistics in study-relevant variables across the four experimental groups. The random allocation of participants to either of the four system conditions did not result in systematic between group differences in APM performance scores (*F*_3,283_ = 1.43, part. η^2^ = .02).

By way of a manipulation check regarding our attempt to ensure sufficient variability in the “preparedness” for the control task, we looked at between group differences in acquired knowledge across systems and across information conditions. As expected, the acquisition of system knowledge differs across the four complexity conditions. In systems with low levels of complexity (i.e., low number of variables, low numbers of relations, low number of dependencies) such as system I, more knowledge was acquired overall compared to in systems with higher levels of complexity (main effect system: *F*_3,275_ = 94.60, part. η^2^ = .51). As intended, the manipulation of the amount of information given to problem solvers had systematic effects on their system knowledge (main effect information: *F*_2,275_ = 5.78, part. η^2^ = .04), with the complete information group acquiring the most and the no-information group the least amount of accurate knowledge. This effect did not differ across the different systems (interaction effect: *F*_6,275_ = 0.79, part. η^2^ = .02) suggesting that the experimental variation of “dosing information” has helped to mitigate effects of confounding knowledge and system complexity. 

The three systems differ from the reference system in specific characteristics that are potentially relevant to complexity. The regression analysis employed to contrast systems I, III and IV against the reference system II revealed that performance scores obtained for system I and IV differed significantly from system II but did not for system III (β_II vs. I_ = .65; β_II vs. III_ = −.06, β_II vs. IV_ = .13). This result pattern stands in contrast to what would have been expected if numbers of variables (NoV) were a system feature relevant to complexity. It also does not align with the prediction that is based on the assumption that the number of relationships (NoR) is a substantial contributor to the complexity of a control task. The obtained result pattern has the best fit—albeit not a perfect one—with the constellation that was predicted based on the proposed complexity framework, which suggested the number of dependencies (NoD) as the complexity-relevant system characteristic. The only deviation relates to the contrast between system II and IV. As system IV has a higher number of dependencies (NoD) than system II we would have predicted higher levels of difficulty (i.e., lower performance scores) controlling it. The result, however, suggests that controlling system IV was easier than system II. This outcome was not predicted under any perspective (see Table 2). 

## 4. Discussion

The aim of this research was to contribute to the ongoing discussion of what constitutes complexity in the context of complex problem solving (CPS). As have done others, we argue that the remaining conceptual vagueness regarding the central term in CPS, namely complexity, is a major hindrance to a meaningful integration of empirical findings and conceptual insights obtained across the still very prolific research landscape in complex problem solving. 

We started this paper with a brief reflection on the three-dimensional variable space of Person, Task and Situation in which experimental research in psychology, and therefore most of the CPS research takes place. This reflection gave a basic orientation for where to look for potential sources of complexity. In this context we emphasized the importance of distinguishing between task and situation as the circumstances in which a task has to be performed is likely to affect the complexity of a task. This stance is derived from more recent discussions in relation to cognitive load theory [34,35]. Following arguments brought forward by Wood (1986) [17], we also pointed out that a task can be conceptualized in at least two ways, one of which is seeing a task as a set of required (cognitive) behaviours; another is in terms of the physical attributes of the stimuli used (task qua task) in a task. In relation to CPS the former reflects on the fact that problem solvers can be given different instructions what to do (e.g., exploring a system to find out its underlying causal structure vs. controlling a system by reaching certain goal states), whilst the latter brings the complex, dynamic system with its concrete features into the focus of conceptual attention. 

Traditionally complexity discussions in CPS research almost exclusively concentrate on quantities of certain system features such as variables and relationships between those variables. The complexity framework proposed in this paper posits that (a) the task as behaviour requirement determines which system and situational features contribute to the complexity of the task, (b) that what contributes to the complexity of a task (as behaviour requirement) are units of information or information cues that need to be processed in parallel, and (c) that individual differences in coping with these complexities result in performance scores which are indicative of the individual level of difficulty problem solvers experience when dealing with the complexity of the cognitive demands as they are imposed upon them under the given situational circumstances.

One consequence of (a) is that system features such as number of variables cannot universally claim to be contributors to complexity, another consequence is that situational characteristics, such as the semantic embedment of a system or the user interface, do also contribute to the complexity of a task (this might often happen in an unintended way). A consequence of (b) is that a merely accumulative perspective on system features (e.g., the total number of relationships) is likely to be misleading. A consequence of (c) is that complexity and difficulty are different concepts and should therefore not be used interchangeably. 

From this complexity framework we then derived specific expectations regarding the relevancy of a set of selected system characteristics as they are frequently discussed as potential sources of complexity. These expectations then were tested using four different complex, dynamic systems where we kept the situational characteristics (e.g., same interface, same semantic embedment) and the task (i.e., controlling the system, bridging the same start-goal discrepancy the output variables) the same. The results show that both the number of variables in a system and the number of relationships in a system do not predict control performance in the pattern predicted by the complexity framework. This suggests that their role as complexity contributors in the context of a control task in CPS is rather limited. This is not to say that NoV is unrelated to complexity. NoR, NoE, and NoD are all functionally linked to NoV, i.e., without variables there cannot be relationships, effects or dependencies. What we can conclude, however, is that NoV as such is most likely a too crude an indicator of complexity and that—depending on the instructed task and the situation—certain qualities of how variables are interconnected are the drivers of complexity. After all, dependencies are a special quality of relationships (which exist between variables) within a complex, dynamic system. This reminds us that complexity is (also) a *qualitative* phenomenon, a notion that can easily be neglected from a purely psychometric point of view with its inherent drive for quantifications (i.e., counting the number of variables). It also underlines the necessity of differentiating between complexity and difficulty.

From a “number of dependencies (NoD)” perspective, the results show for two of the three contrasts effects as predicted by the complexity framework. For the third contrast results indicate—rather unexpectedly—that whilst controlling for knowledge, problem solvers were able to control a system that comprised a dependency of 3 more successfully than those who dealt with a system with a dependency of 2. As a post hoc conjecture, we could speculate that the system with NoD = 3 could be brought sufficiently close to the target values with a causal model that only considers the dependency of “U” from “A” and itself, the dependency of “V” from “A” and itself, and the dependency of “W” from “C” and itself. Such a model would allow the problem solver to successfully circumvent the cognitive demands of dealing the three dependencies of output “U”, making the control task for this system in this instance less complex (i.e., reducing it to a NoD = 2 system). This might be an example of a situation where the whole is less than the sum of its parts. Such post hoc speculations, which could conveniently be framed as a case in point regarding the abovementioned dissociation between complexity and difficulty, need, of course, to be systematically tested. This could be realised, for instance, by using different start-goal discrepancies when asking the system to be controlled.

## 5. Implications and Recommendations

Back to the main objective that underlies this paper, i.e., to encourage future studies, either by collecting new data or by revisiting existing datasets if they have been collected under suitable designs, to refine the proposed complexity framework. In this spirit and as a further facilitation of this intent we now provide a set of lemmata that are based in the proposed complexity framework and are meant as orientation for future studies. 

Complexity in CPS can be sub-categorised into three main facets: detectability, explorability and controllability. Detectability is relevant in the context of knowledge acquisition and refers to the complexity of identifying a causal link between variables. It can be operationalized as the observable size of the effect the maximum intervention input will have on any one of the linked outputs. Low detectability of a causal link exists when changes in the output variables are rather subtle. Arguably, time delays in effects, even if their absolute effect is sufficiently large, are likely to cause low detectability of such links. In line with the tenet that information that needs to be processed in parallel is complexity relevant, it should be the lowest detectability of any relationship within a system that determines its explorability. Another factor that is expected to contribute to the explorability of a system (i.e., a task qua task) is the relative number of effects, which could also be expressed as the density of a system. 

Controllability refers to the complexity of controlling the system, which is determined by the information cues that have to be processed in parallel in order to reach the prescribed goal states in the output variables. As input decisions have to be made sequentially, the maximum number of dependencies that need to be considered for any individual output variable constitutes the complexity that determines the controllability of a given system. The higher the maximum number of dependencies (NoD) for any given output variable the more complex the task of controlling the system and the lower its controllability. The results of the presented study lend support to this lemma. Controllability also depends on the start goal discrepancy as reaching some goal states could allow to ignore some of the dependencies (i.e., reducing complexity by dealing with a reduced NoD —as the result patter in relation to system IV seem to suggest). As different start-goal discrepancies might require different cognitive acts that in and of themselves differ in their sensitivity towards certain system characteristics (NoD, for instance), both start values and goal values—as situational characteristics—are expected to be also critical to the complexity of a control task. Arguably, controllability is likely to be affected negatively by dependencies of a non-linear nature. 

Explorability can be low whilst controllability, i.e., the complexity of reaching the set goal states, could be high. An example for such a situation is where low impact relationships (i.e., those with low detectability) could technically “be ignored” (or remain unknown) when controlling the system. Whilst the score for acquired causal knowledge might subsequently be low (i.e., incomplete knowledge) system control performance on the other hand might be deemed acceptable. As another example, side effects (i.e., links between output variables, [2]) especially in co-existence with autoregressive dependencies of the linked outputs (i.e., eigendynamic), intrinsically have extremely low detectabilities. Asking problem solvers to explore systems with such characteristics would potentially allow studying system control with (necessarily) incomplete knowledge. We are unsure, however, whether this has always been the intention in respective studies

Given the diversity captured under this umbrella term, the proposed complexity framework could prove useful, for instance, in reflections on similarities and differences between various approaches to CPS. The current debate around whether less is more [48], or indeed just less [54] or simply not enough [55], or whether more ends up being too much [56] might be one example. A discussion of advantages and disadvantages of “multiple or minimal complex system” approaches (MCS) in comparison to what is now called “classical CPS”, with the system TAILORSHOP as its poster child, could be informed by reflections grounded in the complexity framework. For instance, the presence or absence of a knowledge acquisition phase, as a variation in situational characteristics, renders controlling the system a very different task. As we have pointed out, different tasks result in different information cues being relevant to their successful execution with consequences regarding complexity and difficulty, and most importantly, the link between the two. In cases were validity is the focus of such “less vs. more discussions” a question has to be addressed first: What do we expect scores obtained in performing different CPS tasks, under different situational conditions and subsequently with different complexities to be valid of? Ignoring such potentially rather fundamental differences when discussing differences in correlations to other measures might create a situation that resembles the proverbial comparison of pomaceous with similarly prominent citrus fruits. 

The proposed framework provides a foundation for the necessary distinction between complexity and difficulty. Difficulty is a concept grounded in psychometrics (i.e., the proportion of successful responses), whilst complexity is primarily a cognitive concept that comprises the interplay of the characteristics of the stimuli, the situation, and the task. Difficulty reflects the level of success of problem solvers (with their inter-individual differences) in dealing with complexity. Psychometric attempts to measure CPS need to be underpinned by a more cognition-based understanding of what is intended to be measured. Difficulty—as a psychometric concept—is of limited explanatory value conceptually. Difficulty estimates provide a descriptive account of some items being answered correctly by proportionally less individuals than other items, which justifies labelling them as being more difficult. If we were now interested in finding out *why* these items are more difficult than others—a question that (cognitive) psychologists are likely to ask—a psychometric position leaves us with the circular reference to the lower proportion of correct answers these item tend to attract. A way of escaping this tautology is to look for potential causes that lie within features of the task, i.e., the stimuli used and the instructions given, and the circumstances under which the task has to be performed (i.e., the situation). The proposed framework is an attempt to structure such efforts and to eventually allow us to move from predominantly ex post facto speculations to truly ex ante predictions. In short, with the proposed framework we intend to facilitate a relocation of CPS from a predominantly psychometric dominion back into the realm of cognitive science, which arguably is the home of the human intellect (at least conceptually). 

The study presented here is not meant to be perceived as a comprehensive test of the introduced framework. We do not claim to have given the ultimate and universally valid answer to the question whether number of dependencies (NoD) is the, or even a reliable complexity component for system control tasks. However, based on the results we are tempted to allocate less confidence into the number of variables (NoV) and/or number of relationships (NoR) in this regard. And this is how this paper should be perceived, namely as an encouragement to reconsider the allocation of confidence in various system features’ relevance to complexity. As a result, it will hopefully take some features out of the focus and might bring in others, some of which might not have been considered yet. We believe that the proposed complexity framework is specific enough to allow for deriving testable hypotheses (see the analyses in this paper as an example), yet open enough to allow modifications and refinements. We wish to invite conceptual and empirical contributions to further develop and refine a common framework that considers the interplay of the person, the task and the situation and has complexity at its conceptual core. This, so we have argued, is particularly pertinent to a research paradigm that carries complexity in its label. We are optimistic that efforts to this end will potentially allow us to better integrate research findings from existing and future studies on complex problem solving.

## Figures and Tables

**Table 1:** Operationalized expectations regarding planned contrasts for each of the potential complexity indicators across the planned contrasts (comparisons in terms of absolute values in parentheses).

System Comparisons	Expectations		
	Number of Variables (NoV)	Number of Relationships (NoR)	Number of Dependencies (NoD)
II vs. I	β_2_ = 0 (6 vs. 6)	β_2_ > 0 (6 vs. 3)	β_2_ > 0 (2 vs. 1)
II vs. III	β_3_ ≪ 0 (6 vs. 12)	β_3_ ≪ 0 (6 vs. 12)	β_3_ = 0 (2 vs. 2)
II vs. IV	β_4_ = 0 (6 vs. 6)	β_4_ < 0 (6 vs. 7)	β_4_ < 0 (2 vs. 3)

**Table 2:** Descriptive statistics.

Conditions	*N*	APM-20	Knowledge	Control
System I	69	62.4 (16.75)	97.8 (7.78)	90.3 (9.81)
System II	71	62.4 (15.74)	50.6 (18.81)	57.1 (9.21)
System III	71	61.3 (16.92)	63.4 (21.57)	56.5 (13.07)
System IV	76	57.6 (16.70)	52.9 (23.48)	62.5 (9.86)

## Footnotes

1 The parallels to the four different uses of CPS, as they have been discussed above, seem not incidental.

2 The ideal values for each input variable, i.e., the intervention that would result in the system exactly reaching the goal state, were calculated by using the values of the output variables on the previous trial and the goal values for the output variables to solve the set of linear equations underlying the system. As the range of possible input values is restricted for the system used in this study (i.e., between −10 and 10), it might not be possible to bring the system into the goal state by a single intervention. In cases as these, i.e., when the ideal values fall outside this range, the values were adjusted to the nearest possible values, which then constituted the optimal values. In cases when the ideal values are within the range of possible inputs, the ideal values were used as the optimal input.

## Appendix A

System I

*U_t _*_+ 1_ = 1.0*U_t_* + 0.8*A* + 0.0*B* + 0.0*C*

*V_t _*_+ 1_ = 1.0*V_t_* + 0.0*A* + 1.6*B* + 0.0*C*

*W_t _*_+ 1_ = 1.0*W_t_* + 0.0*A* + 0.0*B* − 1.0*C*

System II

*U_t _*_+ 1_ = 1.0*U_t_*+ 0.8*A* + 0.8*B* + 0.0*C*

*V_t _*_+ 1_ = 0.8*V_t_* + 1.6*A* + 0.0*B* + 0.0*C*

*W_t _*_+ 1_ = 1.2*W_t_* + 0.0*A* + 0.0*B* − 1.0*C*

System III

*U_t _*_+ 1_ = 1.0*U_t_*+ 0.8*A* + 0.8*B* + 0.0*C*

*V_t_*_ + 1_ = 0.8*V_t_* + 1.6*A* + 0.0*B* + 0.0*C*

*W_t_*_ + 1_ = 1.2*W_t_* + 0.0*A* + 0.0*B* − 1.0*C*

*X_t_*_ + 1_ = 1.0*X_t_* + 0.8*D* + 0.8*E* + 0.0*F*

*Y_t_*_ + 1_ = 0.8*Y_t_* + 1.6*D* + 0.0*E* + 0.0*F*

*Z_t_*_ + 1_ = 1.2*Z_t_* + 0.0*D* + 0.0*E* − 1.0*F*

System IV

*U_t_*_ + 1_ = 0.8*U_t_*+ 0.8*A* + 0.8*B* + 0.0*C*

*V_t_*_ + 1_ = 0.8*V_t_* + 1.6*A* + 0.0*B* + 0.0*C*

*W_t_*_ + 1_ = 1.2*W_t_* + 0.0*A* + 0.0*B* − 1.0*C*
